# *GClnc1* as an emerging oncogenic regulator with mechanistic insights and clinical implications in human cancers

**DOI:** 10.1016/j.isci.2026.117935

**Published:** 2026-10-16

**Authors:** Qing Dong, Yixin He, Rui Yu, Xiaochang Gong, Jingao Li, Fangteng Liu

**Affiliations:** 1 Department of Radiation Oncology, Jiangxi Cancer Hospital and Institute, The Second Affiliated Hospital of Nanchang Medical College, Nanchang 330029, Jiangxi, China; 2 NHC Key Lab of Personalized Diagnosis and Treatment of Nasopharyngeal Carcinoma, Jiangxi Cancer Hospital and Institute, The Second Affiliated Hospital of Nanchang Medical College, Nanchang 330029, Jiangxi, China; 3 Fourth School of Clinical Medicine, Nanchang University, Nanchang 330006, Jiangxi, China; 4 Department of Gastrointestinal Surgery, The Second Affiliated Hospital, Jiangxi Medical College, Nanchang University, Nanchang 330008, Jiangxi, China

**Keywords:** GClnc1, regulatory mechanisms, prognostic prediction, diagnostic value, therapeutic target

## Abstract

Gastric cancer-related long non-coding RNA1 (*GClnc1*) is an emerging oncogenic regulator in multiple malignancies, including gastric, colorectal, ovarian, and bladder cancers, as well as osteosarcoma. It promotes proliferation, inhibits apoptosis, and enhances drug resistance and metastasis. Mechanistically, *GClnc1* dysregulates key cancer-related regulators and pathways, such as p53, MYC, NF-κB, and the SIRT3/SOD2 axis. Clinically, *GClnc1* shows diagnostic and prognostic potential across cancers, with aberrant expression in tissues and altered circulating levels. Notably, exosomal *GClnc1* serves as a circulating biomarker for early gastric cancer detection and predicts response to fluorouracil-based chemotherapy. This review summarizes current evidence on *GClnc1*’s regulatory mechanisms and discusses its translational applications. *GClnc1* may serve as a multi-cancer biomarker and a promising therapeutic target.

## Graphical abstract

**Figure:**
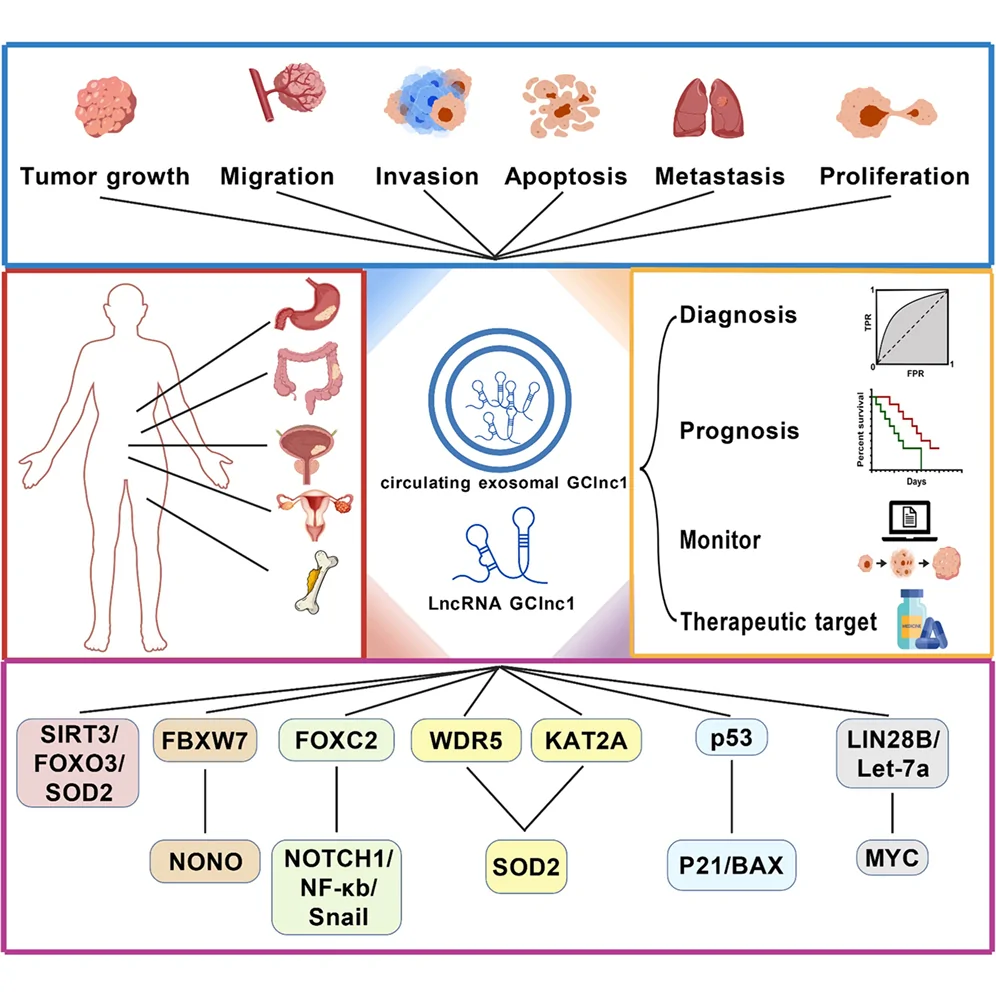

## Teaser

Cancer; Cell biology

## Introduction

Cancer remains a major global health challenge. Approximately 20 million new cancer cases and nearly 10 million cancer deaths occurred worldwide in 2024.^1^ Gastrointestinal (GI) cancers account for a substantial portion of this burden, representing 26% of all cancer cases and 35% of cancer-related deaths in 2018.^2^ Despite advances in treatment, the prognosis for many patients with GI cancers remains poor; for example, in gastric cancer (GC), most recurrences occur within the first two to three years after curative resection,^3^^,^^4^^,^^5^ contributing to unfavorable long-term survival. Because early-stage cancers are frequently asymptomatic or present with nonspecific symptoms, a considerable proportion of cancer patients are still diagnosed at locally advanced or metastatic stages.^6^ Despite advances in surgery, radiotherapy, chemotherapy, and targeted therapies,^7^^,^^8^^,^^9^ there remains an unmet need for better tools to guide treatment decisions, particularly the identification of novel diagnostic and prognostic biomarkers.

Long non-coding RNAs (lncRNAs), defined as transcripts longer than 200 nucleotides with limited protein-coding potential, have emerged as critical regulators in various biological processes and diseases.^10^^,^^11^^,^^12^^,^^13^^,^^14^ Accumulating evidence has demonstrated that lncRNAs play significant roles in tumorigenesis by modulating the expression or activity of oncogenes, tumor suppressors, and key signaling pathways.^15^^,^^16^^,^^17^ Among these, the lncRNA *GClnc1* (gastric cancer-related lncRNA 1, BC041951) was first identified in 2016 by Sun et al.^18^ through whole-genome lncRNA expression profiling in GC tissues. This 2,155-nucleotide transcript, located on chromosome 6, is significantly upregulated in various malignancies and correlates with larger tumor size, metastasis, and unfavorable prognosis.^18^^,^^19^^,^^20^^,^^21^ Mechanistically, GClnc1 promotes tumor progression—including proliferation, invasion, migration, and chemotherapy resistance—by modulating key molecules and pathways such as WDR5/KAT2A, MYC, p53, and the SIRT3/FOXO3/SOD2 axis.^18^^,^^19^^,^^20^^,^^21^

In this work, we provide a comprehensive overview of the aberrant expression and oncogenic roles of *GClnc1* across multiple cancer types, with a focus on its molecular mechanisms of action. Furthermore, we highlight the clinical potential of *GClnc1* as a promising biomarker for early diagnosis, prognosis prediction, and monitoring of therapeutic efficacy, particularly in its circulating exosomal form.

## The role and mechanisms of *GClnc1* in tumor development

The involvement of lncRNA *GClnc1* in various cancers has been investigated *in vitro* and *in vivo*, as summarized in Table 1. Studies have confirmed that *GClnc1* expression is significantly elevated in multiple tumor types compared to corresponding normal cells.^19^^,^^23^^,^^24^ This lncRNA is predominantly localized in the nucleus (Table 1). Functionally, *GClnc1* acts as an oncogene across diverse malignancies (Figure 1), promoting cell proliferation, migration, invasion, tumor growth, and metastasis, while inhibiting apoptosis.^18^^,^^19^^,^^22^^,^^23^^,^^24^^,^^25^ Silencing *GClnc1* expression significantly reduces the migration and invasion capabilities of tumor cells.^23^

**Table 1:** Summary of studies investigating the regulatory roles of lncRNA *GClnc1* in human cancers

Cancer types	Experi-ments	Cell lines	Subcellular localization	Animal models	Functions	Mechanistic response	Roles	Journal
Gastric cancer^18^	*in vitro* and *in vivo*	GES-1, MKN45, AGS, BGC823, SGC7901, MGC803	cell nucleus	4-week-old male BALB/c nude mice	cell proliferation, invasion, chemoresistance, tumor growth, and metastasis	*GClnc1* acts as a scaffold to recruit the WDR5 and KAT2A complex and modify the transcription of target genes	oncogenic	Cancer Discov.
Bladder cancer^19^	*in vitro* and *in vivo*	T24, 5637, UM-UC-3, J82, SV40	cell nucleus	male immune-deficient BALB/c nude mice (5–6 weeks old),10 male BALB/c nude mice (5–6 weeks old)	cell proliferation, invasion, tumor growth, and metastasis	*GClnc1* binds to LIN28B and promotes the expression of myelocytomatosis proto-oncogene through the LIN28B/let-7a/MYC pathway	oncogenic	FASEB J.
Colorectal cancer^22^	*in vitro*	SW620, HCT116, NCM640	cell nucleus	_	cell viability and proliferation	*GClnc1* reduces the expression of p21 as well as BAX via p53 signaling pathway, thereby promoting the progression of colorectal cancer	oncogenic	Eur Rev Med Pharmacol Sci.
Ovarian cancer^23^	*in vitro* and *in vivo*	IOSE80, SKOV3, A2780, DOV13, OVC1, OV90	cell nucleus	NOD/SCID mice (20–22 weeks old)	cell viability, proliferation, apoptosis, EMT, migration, invasion, tumor growth, and metastasis	*GClnc1* knockdown suppresses progression of epithelial ovarian cancer by recruiting FOXC2 to disrupt the NOTCH1/NF-κB/Snail pathway	oncogenic	Exp Cell Res.
Ovarian cancer^20^	*in vitro*	ES-2, T1074	cell nucleus	_	cell proliferation and migration	*GClnc1* regulates p53 signaling pathway	oncogenic	Eur J Histochem.
Osteosarcoma^24^	*in vitro* and *in vivo*	hFOB, 293T, MG-63, HOS, U2OS, 143B	cell nucleus	6-week-old male athymic nude mice (BALB/c background)	cell proliferation and tumor growth	*GClnc1* promotes tumorigenesis in osteosarcoma by inhibiting p53 signaling	oncogenic	Biochem Biophys Res Commun.
Osteosarcoma^25^	*in vitro* and *in vivo*	143B, U2OS, U2R, MG63, 293T	cell nucleus	nude mice (3–4 weeks old)	cell proliferation, migration, invasion, apoptosis, and tumor growth	*GClnc1* promotes osteosarcoma progression by stabilizing NONO and blocking FBXW7-mediated ubiquitination	oncogenic	BMC Cancer
Gastric cancer^21^	*in vitro*	SGC7901	cell nucleus	_	cell proliferation and migration	*GCInc1* promotes the proliferation and migration of gastric cancer cell SGC7901 through the SIRT3/FOXO3/SOD2 signaling pathway	oncogenic	Journal of Third Military Medical University

“–”: there is no relevant data in the study.

**Figure 1:**
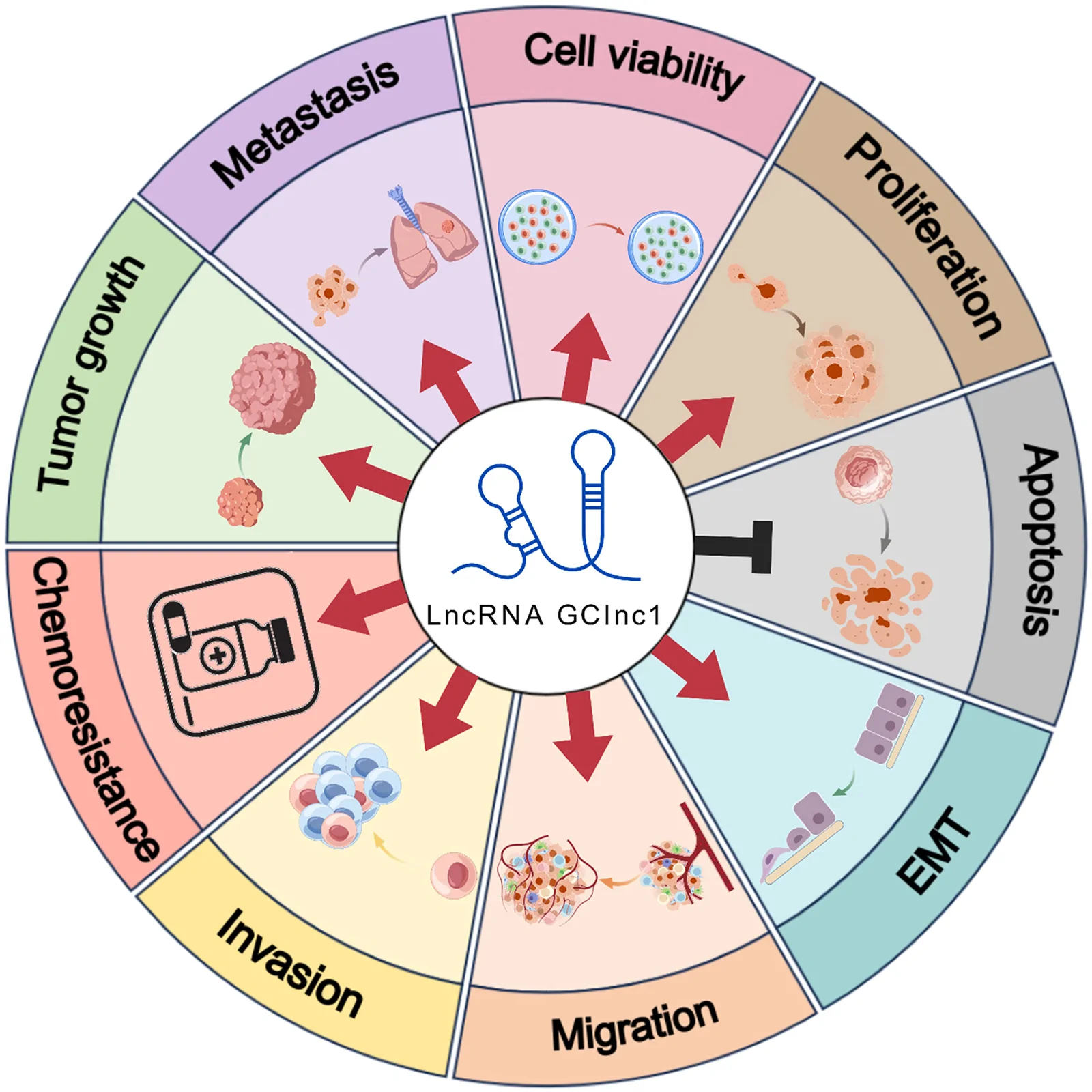
The functions of *GClnc1* in tumorigenesis and progression *GClnc1* overexpression drives proliferation, invasion, migration, and tumor growth, while inhibiting apoptosis and promoting metastasis.

*GClnc1* exerts its oncogenic functions through three archetypal lncRNA mechanisms—guidance, decoy, and scaffolding—in a context-dependent manner. As a guide molecule, *GClnc1* directs protein partners to specific genomic loci: in bladder cancer, it binds LIN28B to promote MYC expression via the LIN28B/let-7a/MYC axis^19^; in epithelial ovarian cancer (EOC), it recruits FOXC2 to activate *NOTCH1* transcription, subsequently triggering the NF-κB/Snail signaling pathway^23^; and in colorectal cancer (CRC), it suppresses p21 and BAX expression through the p53 signaling pathway.^22^ As a decoy, GClnc1 promotes GC progression by upregulating the SIRT3/FOXO3/SOD2 axis.^21^ As a scaffold, *GClnc1* brings together WDR5 and KAT2A complexes to modify histone marks on target genes such as *SOD2*, thereby altering the biological characteristics of GC cells.^18^ The regulatory mechanisms of *GClnc1* are illustrated in Figure 2. In the following sections, we delve into the specific functions and regulatory mechanisms of lncRNA *GClnc1* across various tumor types.

**Figure 2:**
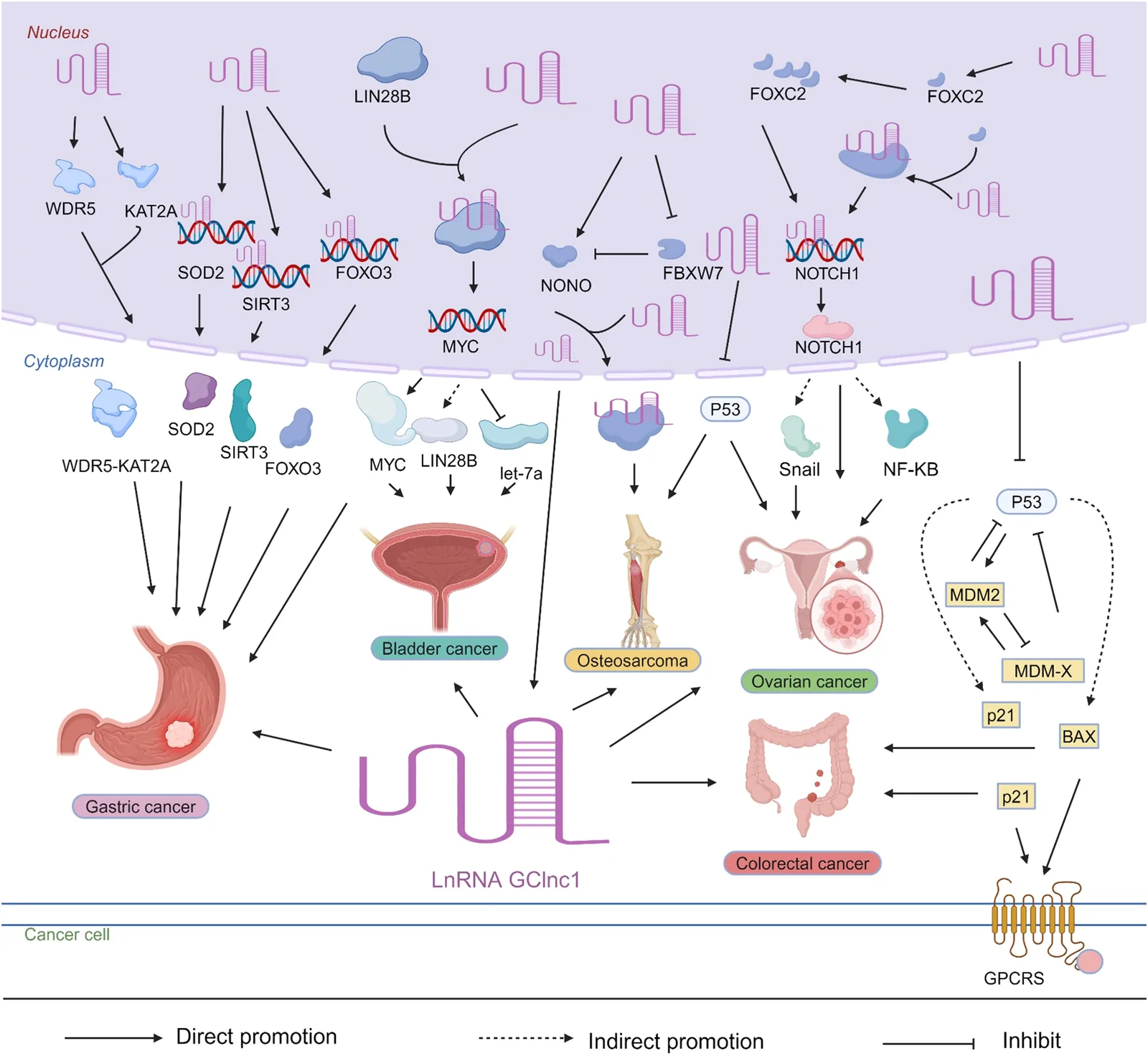
The specific mechanism by which *GClnc1* acts on cancers *GClnc1* regulates cancer progression through multiple mechanisms. In gastric cancer, *GClnc1* forms a complex with WDR5 and KAT2A and increases the levels of *SIRT3*, *FOXO3*, and *SOD2* transcripts and their corresponding proteins. It also enhances MYC expression by modulating the LIN28B/let-7a axis. In ovarian cancer, GClnc1 binds FOXC2, upregulates NOTCH1, activates NF-κB/Snail signaling, and inhibits the p53 pathway. Overall, *GClnc1* promotes tumorigenesis across five cancer types through various molecular mechanisms.

### Gastric cancer

GC ranks as the fifth most diagnosed cancer and the fourth leading cause of cancer-related deaths worldwide.^26^ Research indicates that *GClnc1* is significantly overexpressed in GC tissues and functions as a carcinogenic lncRNA.^18^^,^^21^ Sun et al.^18^ revealed that *GClnc1* acts as a modular scaffold that brings together WDR5, an integral component of the histone methyltransferase complex, and KAT2A, a histone acetyltransferase. By recruiting histone-modifying enzymes (specifically H3K4me3 and H3K9ac) to the promoter region of the *SOD2* gene, *GClnc1* activates its transcription, which subsequently promotes GC cell proliferation, invasion, and metastasis.^18^ Additionally, knocking out GClnc1 enhances the sensitivity of BGC823 and MKN45 cells to 5-fluorouracil and cisplatin, suggesting a role in chemotherapy resistance.^18^ Furthermore, high *GClnc1* expression has been associated with lymph node metastasis and *Helicobacter pylori* infection.^27^

Moreover, *GClnc1* promotes the proliferation and migration of GC cell line SGC7901 through the SIRT3/FOXO3/SOD2 signaling pathway.^21^ Overexpression of *GClnc1* increases the mRNA levels of *SIRT3*, *FOXO3*, and *SOD2*, as well as the levels of their corresponding proteins, while silencing *GClnc1* produces the opposite effect.^21^ *GClnc1* enhances mitochondrial antioxidant capacity and reduces ROS accumulation through this axis, thereby inhibiting chemotherapy-induced cell apoptosis.^21^ Silencing SIRT3/FOXO3/SOD2 eliminates the promotive effects of GClnc1 overexpression on cell proliferation and migration,^21^ suggesting that this pathway may serve as a potential therapeutic target for GC.

### Bladder cancer

Bladder cancer is one of the most common cancers of the urinary system worldwide.^26^ *GClnc1* is significantly upregulated in bladder cancer tissues and cell lines, and its expression correlates with low survival rates.^19^ Mechanistically, *GClnc1* promotes MYC expression by binding to LIN28B in the LIN28B/let-7a/MYC axis, blocking let-7a-mediated degradation of *MYC* mRNA and thereby enhancing proliferation, metastasis, and invasion of bladder cancer cells.^19^ *GClnc1* gene knockdown inhibits the growth and metastasis of bladder cancer cells.^19^ Collectively, these findings indicate that *GClnc1* may serve as a potential therapeutic target for bladder cancer.

### Colorectal cancer

CRC ranks as the third most prevalent cancer globally and represents the third leading cause of cancer-related mortality.^26^ *GClnc1* exhibits significant overexpression in CRC tissues.^22^ Mechanistically, *GClnc1* promotes CRC progression by suppressing p21 and BAX expression through the p53 signaling pathway.^22^ Further studies revealed that *GClnc1* forms a nuclear complex with MDM2, accelerating the ubiquitination of p53. This upregulation markedly diminishes p53 luciferase activity and impairs the binding interaction between p53 and p21, thereby reducing p53-mediated regulation of cancer cell proliferation via the BAX/BCL2 pathway.^22^ *GClnc1* silencing results in a substantial increase in BAX and p21 expression.^22^ These findings suggest that *GClnc1* may serve as a novel therapeutic target for CRC patients.

### Ovarian cancer

Ovarian cancer (OC) is a common malignant tumor of the female reproductive system and a leading cause of cancer-related death in women.^26^ *GClnc1* is significantly overexpressed in EOC tissues, and its knockdown inhibits cell viability and promotes apoptosis.^23^ *GClnc1* promotes *NOTCH1* transcription by recruiting FOXC2, enhances chromatin openness of the *NOTCH1* promoter, activates the NF-κB/Snail signaling pathway, and promotes growth, epithelial-mesenchymal transition (EMT), and metastasis of EOC cells.^23^ During this process, *GClnc1* binds to transcription factor FOXC2 in the nucleus, activating *NOTCH1* transcription. NOTCH1 overexpression promotes proliferation and EMT in SKOV3 and OVC1 cells, and activates the NF-κB/Snail pathway.^23^ *GClnc1* knockdown reduces Snail protein expression and inhibits NF-κB p65 nuclear translocation and phosphorylation, while upregulating FOXC2 and NOTCH1 activates Snail expression.^23^ In addition, *GClnc1* affects cell proliferation and migration through the p53 signaling pathway.^20^^,^^24^ Overexpression of *GClnc1* inhibits p53 reporter luciferase activity and significantly reduces *TP53* and *CDKN1A* mRNA levels and p53 and p21 protein levels, thereby suppressing p53 activity.^19^ Further research is essential to elucidate the therapeutic potential of *GClnc1* in OC.

### Osteosarcoma

Osteosarcoma is a common primary malignant bone tumor with limited treatment options and poor prognosis, particularly in children and adolescents.^26^^,^^28^ *GClnc1* is significantly overexpressed in osteosarcoma, and its knockdown suppresses tumor cell growth both *in vitro* and *in vivo*.^24^^,^^25^ Moreover, *GClnc1* overexpression promotes tumorigenicity of osteosarcoma cells *in vivo*.^24^^,^^25^ Mechanistically, *GClnc1* directly binds to p53 and blocks its interaction with the acetyltransferase p300, thereby suppressing p53 acetylation. This inhibition leads to reduced transcriptional activity of p53 and consequently downregulates the expression of its target genes, *CDKN1A* and *BAX*, ultimately promoting osteosarcoma cell growth and proliferation.^24^ In addition, *GClnc1* competitively binds to the WD40 domain of FBXW7, hindering NONO binding to FBXW7 and reducing FBXW7-mediated ubiquitination degradation, thereby regulating NONO stability. Overexpression of NONO reverses the inhibitory effects of *GClnc1* silencing on cell proliferation, migration, and invasion.^25^ Therefore, factors influencing *GClnc1* expression or *GClnc1*-p53 binding may guide the development of new therapeutic strategies for osteosarcoma.

## Clinical value of *GClnc1* across tumors

*GClnc1* has been found to be significantly upregulated in various malignancies, including GC,^18^^,^^29^ osteosarcoma,^24^^,^^25^ CRC,^22^ bladder cancer,^19^ and OC,^20^^,^^23^ and its expression is associated with adverse clinicopathological features and poor prognosis across different cancer types (Table 2). Among these malignancies, *GClnc1* demonstrates important clinical significance in gastric cancer, especially its circulating exosomal form, which exhibits great potential in tumor monitoring, diagnosis, and prognosis prediction (Figure 3). Notably, encapsulated in exosomes, *GClnc1* is protected from degradation and remains stable under RNase exposure, prolonged room temperature storage, and multiple freeze-thaw cycles.^29^^,^^32^ Additionally, its early dynamic changes further support its utility in GC diagnosis and monitoring.^29^^,^^31^ Beyond GC, the utility of circulating exosomal *GClnc1* in other cancers remains largely unexplored. *GClnc1* is upregulated in CRC and bladder cancer and correlates with poor prognosis,^19^^,^^22^ but direct evidence for circulating exosomal *GClnc1* remains lacking. Similarly, *GClnc1*’s oncogenic roles have been reported in OC^23^ and osteosarcoma,^25^ but its presence in circulating exosomes has not been investigated. Systematic multi-cohort studies are needed to evaluate its diagnostic performance across diverse tumor types.

**Table 2:** Summary of the clinical significance of *GClnc1* in cancer

Cancer types	Sample size	Institution or hospital	Expression	Sample type	Clinicopathologic feature	Clinical prognosis	Diagnosis	Biomarker	Journals
Gastric cancer^18^	165 cases of gastric cancer and adjacent tissues	Shanghai Renji Hospital	up	tissues	pathologic differentiation, vascular invasion, tumor size, and AJCC stage	OS	AUC = 0.732 (combined with AJCC stage)	prognostic and diagnostic	Cancer Discov.
Osteosarcoma^24^	50 pairs of tumor tissues and adjacent non-tumor tissues	No.1 Hospital of Jilin University	up	tissues	tumor size and TNM stage	OS and RFS	_	prognostic	Biochem Biophys Res Commun.
Colorectal cancer^22^	48 pairs of tumor tissues and adjacent normal tissues	The Second Affiliated Hospital of Zhengzhou University	up	tissues	_	OS	_	prognostic	Eur Rev Med Pharmacol Sci.
Bladder cancer^19^	60 pairs of bladder cancer tissues and matched paracarcinoma tissues, 371 bladder cancer patients from TCGA	Peking University Shenzhen Hospital	up	tissues	histological grade and T stage	OS	_	prognostic	FASEB J.
Gastric cancer^29^	522 patients, 85 with precancerous lesions, 219 healthy donor individuals	Chinese People’s Liberation Army General Hospital	up	serum	T stage and N stage	_	AUC = 0.9033 specificity = 85.16% sensitivity = 91.78%	diagnostic	JAMA Surg.
Gastric cancer^27^	80 pairs of cancerous and marginal non-cancerous GC samples tissues	Noor Nejat Hospital	up	tissues	lymph node metastasis, and *H. pylori* infection	_	AUC = 0.8228 sensitivity = 90% specificity = 61.67%	diagnostic	Clin. Lab.
Epithelial ovarian cancer^23^	57 tumor and paired paracancerous tissues	First Hospital of Quanzhou Affiliated to Fujian Medical University	up	tissues	FIGO stage and lymph node metastasis	OS	_	prognostic	Exp Cell Res.
Gastric cancer^30^	981 GC patients	Air Force 986th Hospital, Xijing Hospital, Chinese PLA General Hospital	up	serum	T stage, N stage, and AJCC stage	OS and DFS	_	prognostic	EBioMedicine.
Gastric cancer^31^	888 with GC, 158 with chronic atrophic gastritis, 193 with intestinal metaplasia, 501 healthy donors, and 401 with other gastrointestinal cancers	Air Force 986th Hospital, Xijing Hospital, Chinese People’s Liberation Army General Hospital	up	tissue, plasma	_	_	AUC = 0.9369 sensitivity = 87.42% specificity = 84.82%	diagnostic	Gastroenterology.
Gastric cancer^32^	798 GC patients receiving neoCT	Xijing Hospital, Air Force 986th Hospital, Chinese PLA General Hospital	up	serum	_	OS and DFS	AUC = 0.9586	prognostic and diagnostic	Int J Surg.

“–”: there is no relevant data in the study; GC: gastric cancer; OS: overall survival; RFS: recurrence-free survival; DFS: disease-free survival; AUC: area under the curve.

**Figure 3:**
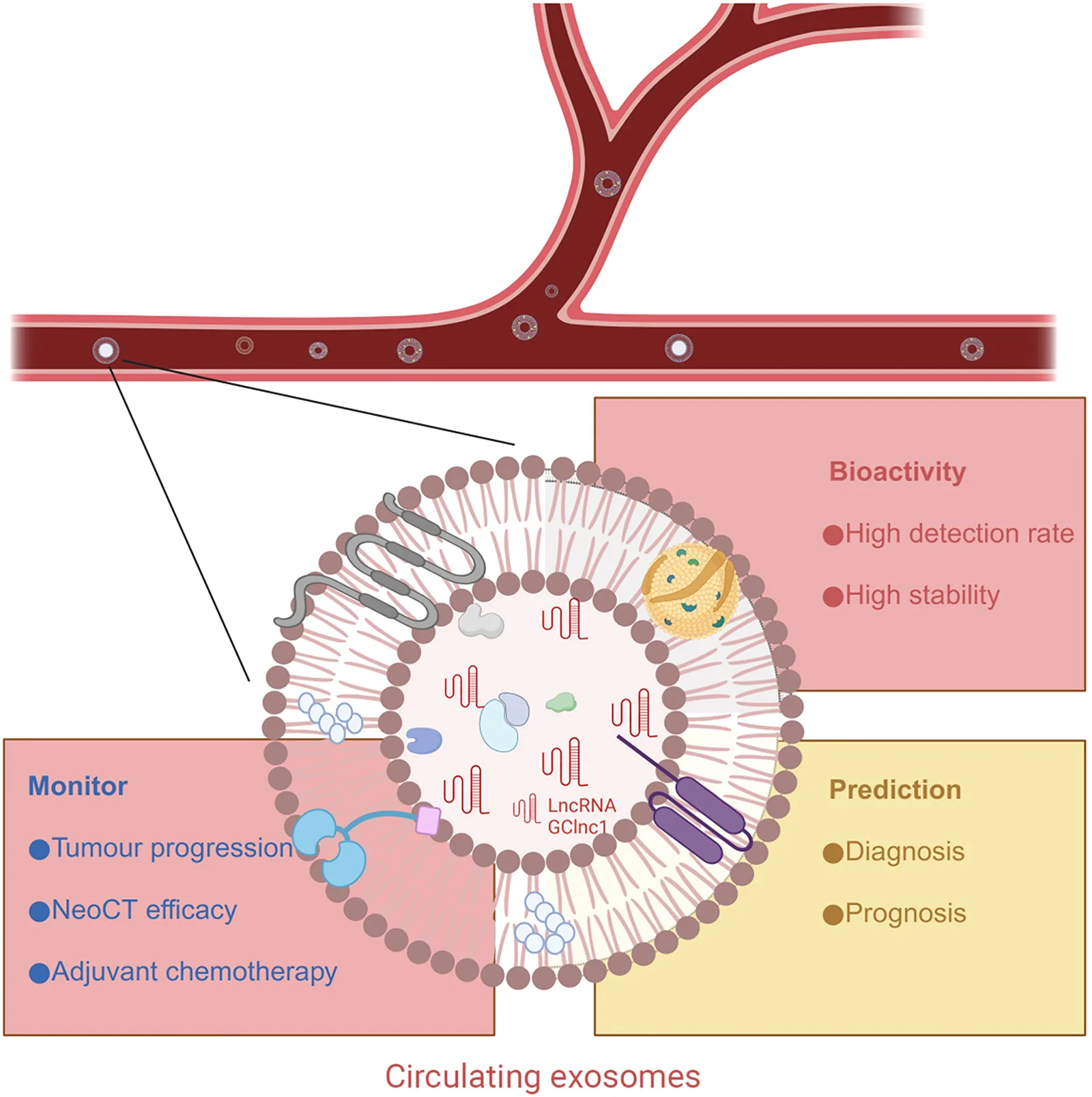
Clinical significance of circulating exosomal *GClnc1* in gastric cancer Circulating exosomal *GClnc1* is stable and detectable, making it a useful biomarker for cancer diagnosis, prognosis, and real-time monitoring of tumor progression and chemotherapy response.

### Diagnostic value of *GClnc1* in gastric cancer

#### Tissue-derived *GClnc1*

Research has demonstrated that *GClnc1* is significantly overexpressed in GC tissues.^18^ ROC analysis revealed that the combined predictive model incorporating *GClnc1* expression and AJCC staging exhibited a superior AUC compared to AJCC staging alone (0.732 vs. 0.695).^18^ Additionally, *GClnc1* demonstrated robust diagnostic performance in differentiating GC from normal tissues, with a sensitivity of 90% and specificity of 61.67%.^27^ These findings suggest that tissue-based *GClnc1* assessment could serve as a valuable diagnostic tool for GC.

#### Blood-derived circulating exosomal *GClnc1*

Circulating exosomal *GClnc1* is significantly upregulated in GC patients and shows a strong correlation with tissue *GClnc1* levels, supporting its role as a GC-specific non-invasive biomarker.^29^ Clinical studies have documented a notable decrease in circulating exosomal *GClnc1* levels following surgical intervention, further corroborating its specificity for GC.^31^

Guo et al.^29^ demonstrated that circulating exosomal *GClnc1* levels are significantly elevated in early GC patients compared to healthy controls and increase in a stage-dependent manner from clinical stage I to IV. This suggests its utility for both early detection and disease progression monitoring.^29^ In diagnostic performance evaluation, circulating exosomal *GClnc1* achieved an AUC of 0.9033, significantly outperforming traditional markers including CEA (0.5987), CA72-4 (0.6816), and CA19-9 (0.6482).^29^ Notably, in distinguishing early GC from precancerous lesions (chronic atrophic gastritis and intestinal metaplasia), circulating exosomal *GClnc1* showed remarkable performance (AUC: 0.9369; sensitivity: 87.42%; specificity: 84.82%), substantially surpassing conventional biomarkers.^29^^,^^31^ Furthermore, it demonstrated earlier dynamic changes during GC progression while maintaining optimal diagnostic accuracy, particularly in patients with negative standard biomarker results.^32^ These findings highlight circulating exosomal *GClnc1* as a superior diagnostic tool that could enable earlier and more accurate GC detection.

### Prognostic significance of *GClnc1* in cancers

#### High expression of *GClnc1* in cancer samples correlates with poor prognosis across multiple cancers

Accumulating evidence from multiple studies has demonstrated that *GClnc1* overexpression is prevalent in various malignancies, including GC,^18^^,^^29^^,^^30^ osteosarcoma,^24^^,^^25^ EOC,^23^ CRC,^22^ and bladder cancer,^19^ with its elevated expression consistently correlating with unfavorable clinical outcomes. In GC, elevated *GClnc1* expression has been significantly associated with advanced tumor progression (pathological differentiation and tumor size), aggressive pathological features (vascular invasion and poor differentiation), advanced clinical stages, and reduced overall survival (OS).^18^ Similarly, in osteosarcoma, patients with high *GClnc1* expression exhibited significantly worse OS and disease-free survival (DFS) compared to those with low expression, along with correlations to larger tumor size and advanced TNM staging.^24^ This prognostic significance extends to EOC and CRC, where *GClnc1* overexpression has been independently associated with reduced survival time.^22^^,^^23^ Furthermore, in bladder cancer, *GClnc1* upregulation has been significantly correlated with both decreased OS and higher histological grade.^19^ These findings underscore the potential role of *GClnc1* as a pan-cancer prognostic biomarker.

#### Prognostic utility of circulating exosomal *GClnc1* in gastric cancer

##### *Circulating exosomal GClnc1 levels significantly correlate with OS and DFS in gastric cancer patients*

A multi-cohort, multi-phase study by Song et al.^30^ demonstrated a significant correlation between circulating exosomal *GClnc1* levels and GC prognosis, revealing that patients with lower circulating exosomal *GClnc1* expression exhibited significantly improved OS and DFS compared to those with elevated levels. Furthermore, multivariate Cox proportional hazards regression analysis identified circulating exosomal *GClnc1* as an independent prognostic factor for patients with resectable GC.^30^

##### *Prognostic role of circulating exosomal GClnc1 in predicting chemotherapeutic response in advanced gastric cancer patients*

In current clinical practice for GC management, postoperative adjuvant chemotherapy is routinely recommended for patients with advanced disease.^33^ However, evidence from pivotal clinical trials indicates significant heterogeneity in treatment response, with not all patients deriving clinical benefit from adjuvant chemotherapy.^34^ This underscores the critical need for predictive biomarkers to identify patient subgroups most likely to benefit from adjuvant treatment. Notably, emerging evidence suggests that stage II and III GC patients with low circulating exosomal *GClnc1* expression levels may derive superior clinical benefit from fluorouracil-based adjuvant chemotherapy regimens.^29^

##### *Prognostic significance of circulating exosomal GClnc1 in predicting response to neoadjuvant chemotherapy*

Neoadjuvant chemotherapy (neoCT) followed by radical surgery has emerged as the standard therapeutic approach for advanced GC, with the primary objective of achieving R0 resection and improving OS rates.^35^^,^^36^^,^^37^^,^^38^^,^^39^ In clinical practice, the determination of optimal surgical timing for GC patients undergoing neoCT predominantly relies on radiological imaging assessment. However, early treatment response evaluation is critical to minimize treatment delays in non-responders while reducing unnecessary toxicity in responders. Recent investigation has explored the potential utility of circulating exosomal *GClnc1* as a dynamic monitoring biomarker for neoCT response.^32^ Notably, circulating exosomal *GClnc1* demonstrates earlier dynamic changes compared to conventional gastrointestinal biomarkers during the first cycle of neoCT.^32^ The response patterns of circulating exosomal *GClnc1* show high detection rates and strong correlation with radiological response, enabling earlier clinical decision-making in neoCT management.^32^ Importantly, GC patients who achieved at least a 50% reduction in circulating exosomal *GClnc1* levels during neoCT had significantly improved DFS and OS.^32^

## Discussion and future perspectives

Beyond its well-characterized oncogenic functions (Figure 4), *GClnc1* has emerged as a promising biomarker with significant clinical potential. It demonstrates potential value in tumor staging and prognosis prediction across multiple malignancies,^19^^,^^22^^,^^23^^,^^24^^,^^29^^,^^30^^,^^31^ while circulating exosomal *GClnc1* shows particular promise in GC management,^29^^,^^30^^,^^31^^,^^32^ including early diagnosis, evaluation of therapeutic efficacy, and monitoring of disease progression (Figure 5).

**Figure 4:**
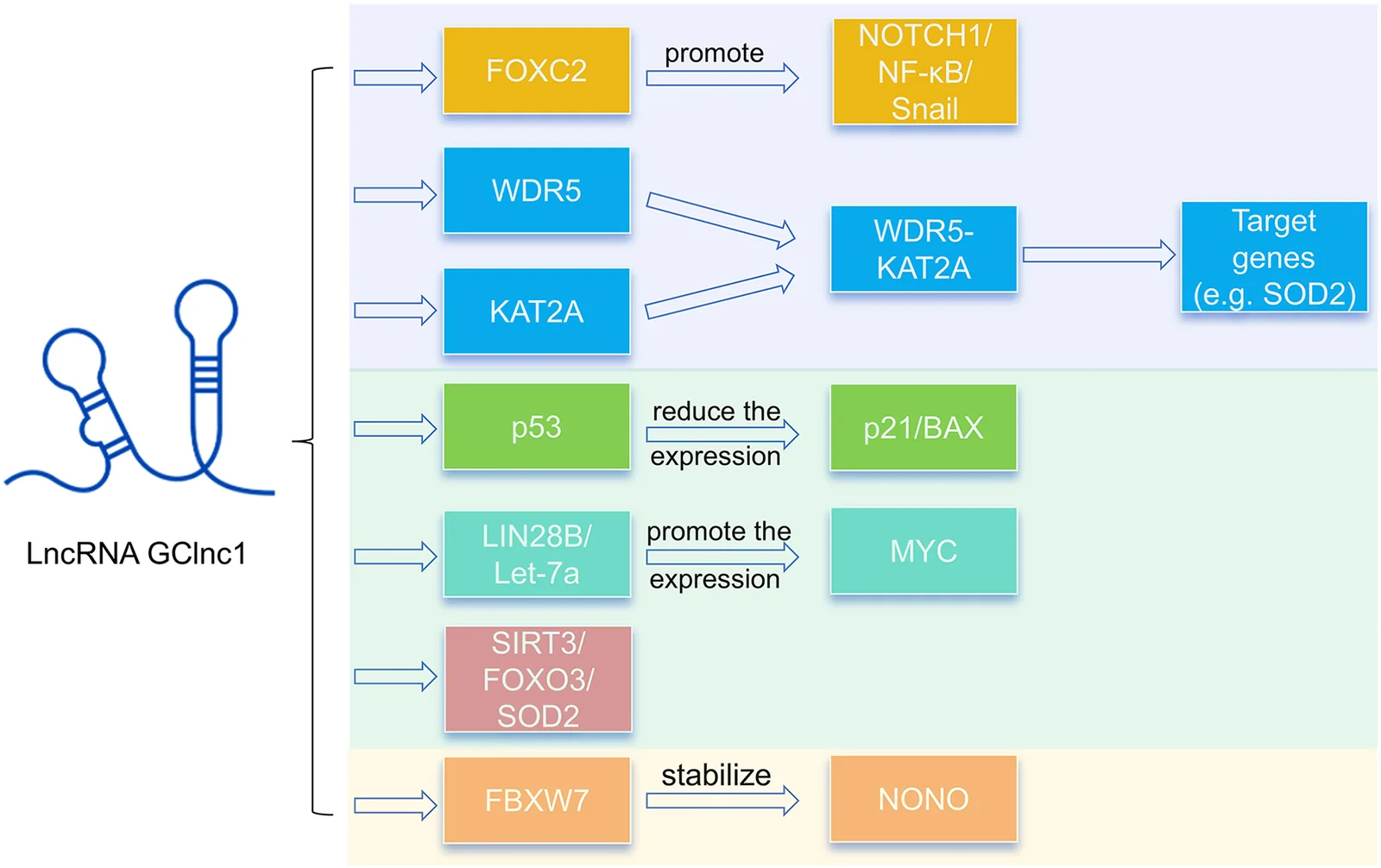
Regulatory mechanisms associated with lncRNA *GClnc1* in cancers *GClnc1* acts through three major modes (color-coded). (1) Epigenetic and transcriptional regulation (blue): recruiting FOXC2 to activate NOTCH1/NF-κB/Snail signaling, and forming a WDR5/KAT2A complex to modulate target gene expression. (2) Signaling pathway modulation (green): inhibiting the p53 pathway (reducing p21/BAX), activating the SIRT3/FOXO3/SOD2 pathway, and enhancing MYC via the LIN28B/let-7a axis. (3) Post-translational regulation (orange): blocking FBXW7-mediated ubiquitination to stabilize NONO.

**Figure 5:**
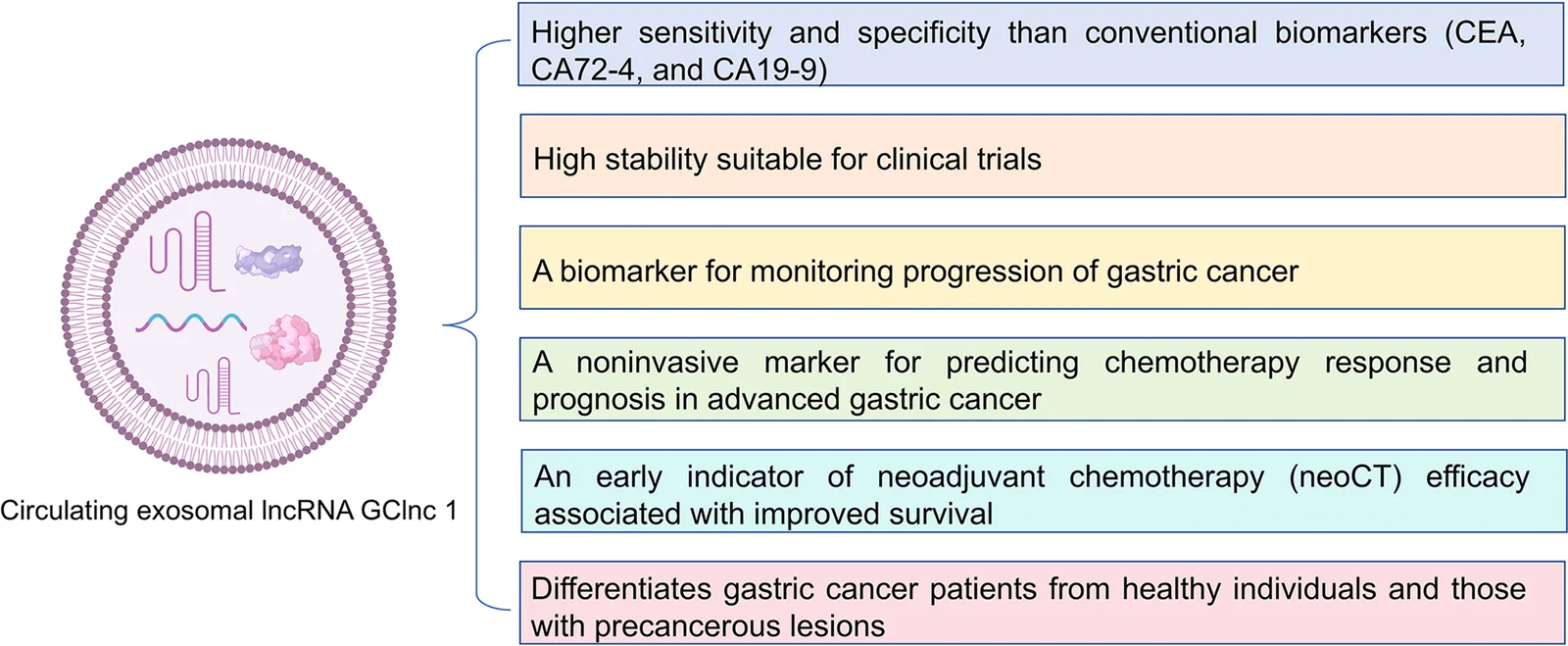
The advantages of circulating extracellular vesicles in gastric cancer Circulating exosomal *GClnc1* shows high diagnostic performance and stability. It can monitor GC progression, predict chemotherapy response and prognosis, and indicate neoCT efficacy linked to improved survival. It also distinguishes GC from healthy individuals and precancerous lesions.

Despite this progress, current research on *GClnc1* faces several limitations. First, although its oncogenic role has been reported in multiple cancers, investigations in other malignancies remain limited, restricting its potential as a pan-cancer biomarker. Second, existing studies have predominantly focused on isolated signaling pathways^20^^,^^22^^,^^24^ or specific gene expression patterns,^18^^,^^27^ with insufficient attention to complex regulatory networks such as ceRNA mechanisms. These gaps hinder both a comprehensive understanding of *GClnc1* and the development of *GClnc1*-targeted therapies.

To address these gaps, future research should expand *GClnc1* investigations to a broader range of solid tumors and hematological malignancies. However, the latter requires careful consideration of the distinct biological features of hematological malignancies. Unlike solid tumors, where *GClnc1* has been consistently detected and shown to regulate signaling pathways mediated by p53,^24^ MYC,^19^ and NF-κB,^23^ hematological malignancies arise from hematopoietic cells with different microenvironments, exosome biogenesis mechanisms, and regulatory networks.^40^^,^^41^^,^^42^ The bone marrow microenvironment and exosome biogenesis in hematopoietic cells differ from those in solid tumor cells.^42^ Therefore, while exploring *GClnc1* in hematological tumors is an important goal, foundational studies are first needed to explore its expression patterns, functional relevance, and mechanistic roles in these hematological malignancies.

Additionally, the clinical application of circulating exosomal *GClnc1* has thus far been confined to GC. Future studies should expand the scope and scale of clinical research to a broader range of tumor types and disease stages. This will enable a comprehensive assessment of *GClnc1*’s diagnostic accuracy and reliability across diverse malignancies.

For clinical translation, several technical challenges should be addressed. The development of more sensitive and specific detection methodologies for quantifying *GClnc1* in patient samples, alongside the refinement of efficient and reliable exosome extraction and purification techniques, will be critical to ensure accuracy and reproducibility. Furthermore, the establishment of standardized testing protocols and rigorous quality control measures will enhance the comparability and reliability of *GClnc1* testing across different laboratory settings. Overcoming these hurdles will be essential for the widespread clinical adoption of *GClnc1* as a robust diagnostic biomarker and, potentially, a therapeutic target.

## Conclusion

*GClnc1* is a multifunctional oncogenic lncRNA. It exhibits potential as a prognostic indicator across multiple cancers, as well as a circulating biomarker for early gastric cancer detection and therapeutic monitoring, while also representing a promising therapeutic target. However, future endeavors should prioritize more in-depth mechanistic studies, multi-cancer validation studies, and large-scale multicenter clinical studies to fully harness its translational potential.

## Acknowledgments

This study was supported by grants to Jingao Li from the Non-profit Central Research Institute Fund of the 10.13039/501100005150Chinese Academy of Medical Sciences (no. 2020-PT320-004) and the Key Program of the Jiangxi Province Key Research and Development Program (no. 20232BBG70025), and to Qing Dong from the Science and Technology Projects of the Health Commission of Jiangxi Province (no. 202211037) and the Scientific Research Fund Project of Jiangxi Cancer Hospital (no. KFJJ2023YB11).

## Author contributions

Q.D.: funding acquisition; supervision; writing – original draft (Introduction, Discussion and future perspectives, and Conclusion); writing – review and editing (whole manuscript). Y.H.: data curation (Table 1); visualization (Figures 2, 3, 4, and 5); writing – original draft (the role and mechanisms of GClnc1 in tumor development and clinical value of GClnc1 across tumors); using Adobe Illustrator and Excel. R.Y.: data curation (Table 2); visualization (Figure 1 and Graphical Abstract); writing – original draft (the role and mechanisms of GClnc1 in tumor development and clinical value of GClnc1 across tumors, jointly with Y.H.); using Adobe Illustrator and Excel. X.G.: Literature analysis and interpretation (figure data); writing – review and editing (critical revision of the role and mechanisms of GClnc1 in tumor development and clinical value of GClnc1 across tumors). J.L.: funding acquisition; Literature analysis and interpretation (table data); writing – review and editing (critical revision of Discussion and future perspectives and Conclusion). F.L.: conceptualization; project administration; supervision; data curation and visualization (assisted in preparation and verification of all figures and tables); writing – original draft (contributed to writing all sections of the manuscript and integrated the final text); writing – review and editing (whole manuscript). All authors have read and approved the final version of the manuscript and agree to be accountable for all aspects of the work.

## Declaration of interests

The authors declare no competing interests.
